# Consciousness as the Temporal Propagation of Information

**DOI:** 10.3389/fnsys.2022.759683

**Published:** 2022-03-23

**Authors:** Daniel Revach, Moti Salti

**Affiliations:** Department of Neuroscience, Ben-Gurion University of the Negev, Be’er Sheva, Israel

**Keywords:** consciousness, information theory, perception, neural correlates of consciousness (NCC), body boundaries, volition, self

## Abstract

Our ability to understand the mind and its relation to the body is highly dependent on the way we define consciousness and the lens through which we study it. We argue that looking at conscious experience from an information-theory perspective can help obtain a unified and parsimonious account of the mind. Today’s dominant models consider consciousness to be a specialized function of the brain characterized by a discrete neural event. Against this background, we consider subjective experience through information theory, presenting consciousness as the propagation of information from the past to the future. We examine through this perspective major characteristics of consciousness. We demonstrate that without any additional assumptions, temporal continuity in perception can explain the emergence of volition, subjectivity, higher order thoughts, and body boundaries. Finally, we discuss the broader implications for the mind-body question and the appeal of embodied cognition.

## Defining and Approaching Consciousness

The mind-body problem is encumbered by the difficulty in describing the conscious mind. While consciousness has been defined in many ways, most scientists and researchers in the field hone in, to varying degrees, on Nagel’s idea of “what it is like” (Nagel, 1974). This formulation of subjective experience is formally referred to as qualia (Block, 2004): perceptions of sounds, smells, pain, and more generally, that private world consisting of mental states like sensations, feelings, and thoughts. They exhibit continuity over time, and can be turned outward at the world or inward at the self (Koch, 2004). The different phenomenological aspects of consciousness are difficult to tie together. It is even more challenging to find common principles that explain all of them, let alone generalize those principles across species and non-biological systems.

To understand the mind-body relationship, current research attempts to identify specific neurological areas and activities correlating with and potentially responsible for consciousness, or the neural correlates of consciousness (NCC) (Crick and Koch, 1990; Boly et al., 2017; Mashour et al., 2020). This approach derives from the idea that consciousness takes place somewhere in the brain and has a particular function that is distinct from other processes (Zeki, 2003; Pereira and Ricke, 2009; Blackmore, 2016). This is nothing new; with his famous declaration “I think, therefore I am,” Descartes brought the relationship between mind and body to center stage (Kenny, 1968; Basile et al., 2010). The French philosopher attributed the mind’s seat to the pineal gland - an idea that while dismissed, shares many similarities with today’s approach. This reductionism breaks down the neural substrate into its most elementary constituents. Working its way down the neurobiological structure, current research aims to find the component or “missing ingredient” (Lamme, 2018) responsible for a conscious representation by distilling it from other processes considered unconscious (see Aru et al. (2012), de Graaf et al. (2012), Sergent and Naccache (2012)). The conscious mechanism or faculty “inside the brain” is considered the fundamental unit of consciousness, conditionally necessary for the generation of awareness, even in isolation. Importantly, the generation of a conscious “percept” or “episode” is viewed as a discrete neural event in space and time (Salti et al., 2019). The unit of consciousness is thus distinguished from the rest of the nervous system and the body. It should invariably correlate with awareness, and cleanly differ from early upstream and late downstream processes. Much like Descartes’ pineal gland, some current candidates are the prefronto-parietal network (Dehaene and Changeux, 2011), thalamus (Ward, 2011), feedback loops in sensory areas (Lamme, 2004), and the posterior cortex (Tononi et al., 2016; for a longer but older list, see Chalmers, 2000). As a result, major debates today are framed in terms of where in the brain is the “seat” of consciousness and when it is activated (for a recent example, see Melloni et al., 2021).

There are several significant limitations with this view of consciousness [for a general review, see (Revach and Salti, 2021)]. According to recent studies, different conditions elicit different NCCs (Melloni et al., 2011). Indeed, many areas and activities in the brain seem to be somehow involved with conscious experience in a distributed fashion, such as the candidates mentioned above and many more (Bisenius et al., 2015). There is an increasing awareness of the importance of spatiotemporal dynamics (He, 2018; Northoff and Lamme, 2020). Furthermore, any NCC identified would be difficult to generalize across species that do not possess the same neural structures. The same problem arises with the reliance on the ability to report events with accuracy, clearly a derivative of human experience, as the standard behavioral index (Seth et al., 2005). Besides, the association between consciousness and reportability has been undermined time and again (Wolfe, 1999; Snodgrass et al., 2004; Chen and Wyble, 2015; Born et al., 2019). Finally, by focusing on the physical properties of conscious experience, a separation is made between phenomenology and mechanism that neglects phenomenology (Seth and Hohwy, 2020). Even if the NCC are to be mapped out, the approach would be hard pressed to explain why these correlates are qualitatively different than other neural activities, i.e., why they alone give rise to phenomenal experience and other aspects of consciousness.

In contrast to the reductionist perspective, consciousness is increasingly viewed as an emergent property (Levine, 2001; Chalmers, 2003). Only animals can so far be identified as conscious systems, yet they can be broken down into nothing more than physical constituents that are ubiquitous across the entire universe. Emergence offers a perspective (Anderson, 1972) that could help overcome the challenges faced today in understanding consciousness. There is a need for a model to explain how conscious experience is produced and what its characteristics are, without relying on derivative properties like brain areas, activity patterns, or specific experiences. A satisfactory theory of consciousness should identify fundamental properties of consciousness.

One such framework that offers an emergent perspective is information theory. Researchers like Karl Friston, Anil Seth, Giulio Tononi and their colleagues (Tononi and Edelman, 1998; Tononi, 2012; Seth and Friston, 2016; Solms and Friston, 2018; Hohwy and Seth, 2020; Seth and Hohwy, 2020) have proposed an information-theoretic perspective. By characterizing the emergence of consciousness from the dynamics between the physical constituents of a conscious system, we might obtain a quantitative rather than qualitative account and identify fundamental properties that are universal (Seth et al., 2006). Such theories emphasize the spatial dynamics of information processing (Tononi, 2012). However, temporal dynamics of information processing have hardly been explored. The time dimension has only recently been receiving attention which has just scratched the surface (He et al., 2010; He, 2018; Winters, 2020; Wiese and Friston, 2021). Based on the observation that conscious experience is continuous across time, Winters (2020) proposes temporal causality as a primary factor driving consciousness. In this article, we adopt the concept of temporal integrity (Krakauer et al., 2020) to present consciousness as the propagation of information across time. The approach we propose is unique in that it attempts to define consciousness with the temporal continuity of perception at its center. We demonstrate how this starting point, using minimal assumptions, leads to the emergence of properties that are associated with consciousness but rarely accounted for in models of consciousness. In this manner our approach suggests a way of unifying fundamental aspects of cognition, neural structures, phenomenology, and subjectivity, while remaining *a priori* unconstrained by any specific subjective (e.g., phenomenological experiences) or objective (e.g., brain regions) properties. Above all, we argue that this view takes us a step closer toward obtaining a parsimonious definition and description of consciousness as well as valuable insight into the mind-body problem.

## Information Theory

Information theory was first formulated by Claude Shannon, based on the concept of entropy developed by Boltzmann and Clausius in the 19th century (Balibrea, 2016). Clausius introduced a definition of work as the transference of thermal energy from one body to another. Entropy accordingly measures the loss in energy from the total available energy for performing work. Later on, in his kinetic theory of gasses, Boltzmann introduced an alternative interpretation of entropy, as the potential disorder in a system. This reframes the topic as the number of possible configurations (microstates) of a system consistent with a particular microstate.

In 1948, Claude Shannon made use of the concept of entropy to measure the informational capacity of a communication channel (Shannon, 1948; Gappmair, 1999). A data string of a given length (a macrostate) is compatible with several sequences of symbols (microstates). During transmission, the target message would be disordered in proportion to the noise in the channel; the more it is disordered, the higher the entropy. Accordingly, entropy quantifies the amount of uncertainty involved in the outcome, i.e., the message received. In contrast, information entropy represents the number of states that can be transmitted from one point to another across a channel, in the face of noise and when efficiently encoded. Information is the average amount of information shared through a channel transmitted from a signaler and a receiver. In this sense, the model identifies and describes causality, or the degree to which the state of a system, i.e., the outcome, derives from another system or a previous state of the same system. A state as such constitutes a specific configuration of the system under observation out of the gamut of the possible configurations it may occupy. Information theory has proven to be highly generalizable, with applications in communication, statistics, computer science, astronomy, linguistics, and genetics (Pierce, 2012).

## Consciousness Through Information Theory

Looking at consciousness through information theory, we propose that consciousness should be viewed as a process across time in which the system transitions from one state to the next. Consciousness is dependent on the propagation of information from the past to the future (Hohwy et al., 2015; Salti et al., 2019). Given nothing but a system, whether it is the brain or the human being as a whole, whose perception of its surroundings is temporally continuous, subjective experience will arise as an interaction history with the environment accumulates.

The approach draws on Process Philosophy (Hartshorne, 1978; Rescher, 2007), emphasizing the dynamical, changing nature of phenomena as the fundamental principle, instead of starting from objects, such as mental states, brain faculties, or percepts and then analyzing their properties and relations. Alternatively, it is the process of becoming rather than being that is made the core of research, as it gives rise to entities we perceive as objects. Information theory lets us contemplate a temporal process by considering that the signaler and receiver can be distinguished through time, where the same system is the signaler in the past and the receiver in the future. In this sense, consciousness is the transmission of information from a system to itself over time. If the information transmitted is high, meaning the entropy is low, this would provide evidence for consciousness.

**FIGURE 1:**
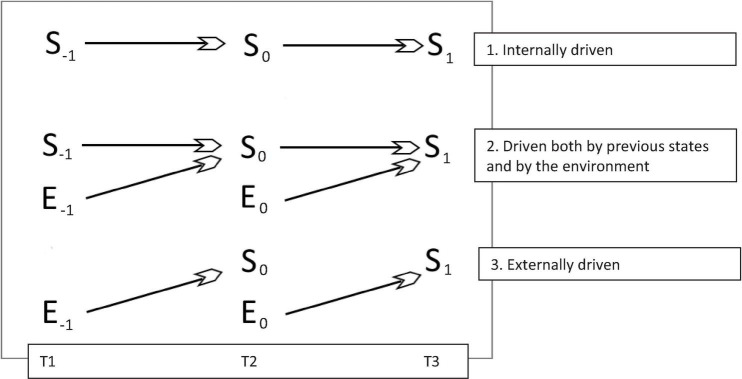
Consciousness emerges in systems as a function of the degree in which futures states are determined by past states. Such a system can be said to be internally or endogenously driven. In flow chart 1, the system (S) best predicts its own future states. By looking at the system at time 0, we can predict what the system will be at time 1, or just the same what it was at time –1. This can allude to cases where the Default Mode Network (DMN) is active, and the mind is turned inward rather than being influenced by the senses. In flow-chart 2, the system is driven both by itself and by the environment (E). The flow of consciousness selectively integrates information from the environment. In flow-chart 3, the system is low in consciousness, and its behavior is entirely by the environment, or can be said to be externally driven. There is no continuous flow of internal information processing.

In this manner, consciousness emerges from an aggregate of elements that are temporally integrated (Winters, 2020). The information maintained by the system is maximal, such that it is differentiated from the environment (Solms and Friston, 2018; Cooke, 2020): external conditions as mediated by perception have less influence over the future state of the system than its previous states. We can say that such a system is endogenously or internally determined (see Figure 1), and therefore highly conscious. In order to know what the future state of the system will be at time t + 1, we can look at the state of the system at time t. In phenomenological terms, this would mean that it is one’s thoughts and sensations that lead to new thoughts and sensations, as well as new behavior, rather than being driven by new input from the environment. The amount of information maintained by the system across time is influenced by the complexity of the system; the more microstates a system can assume, the richer the experience when the variables are determined (Oizumi et al., 2014).

Internal consistency over time despite fluctuations in the environment reflects a reduction of entropy, or uncertainty, and therefore an increase in predictability (Hohwy and Seth, 2020). According to Friston (2010), systems endowed with cognition are defined by the instantiation of a generative model. They calculate the probability of causes given sensory data and priors. Optimizing brain states allows a model or representation of the world where few states are very probable and therefore the outcome is predictable, contrary to a poor model that would have all states equally (im)probable and therefore offer poor predictability.

The relationship between the self and the environment is to a certain extent mediated by consciousness. The generative model is not only a model of the world, but at the same time a model of the self (Ramstead et al., 2018). Minimizing surprise is the same as maximizing the sensory evidence of an agent’s existence. It is precisely the dynamics of conscious experience across time that allow piecing together the system and its counterpart, the environment (Salti et al., 2019).

How does a conscious system generate information, or reduce uncertainty? In order to generate a model of the environment, the system identifies regularities by coarse-graining or compressing time series data (Allefeld et al., 2009; Oizumi et al., 2014). Coarse-graining increases the probability of observing coherent behavior. Coarse-grained slow variables are robust in that they ignore erratic deviations or fluctuations in the data. In this manner they are more regular. These regularities also characterize the conscious system itself: internal regularities make it resistant to influence from the environment, resulting in a high measure of shared information with itself in different time stamps. He and Raichle (2009) identify slow patterns of cortical potentials with consciousness, reflecting the stability and robustness of activity associated with conscious experience.

**FIGURE 2:**
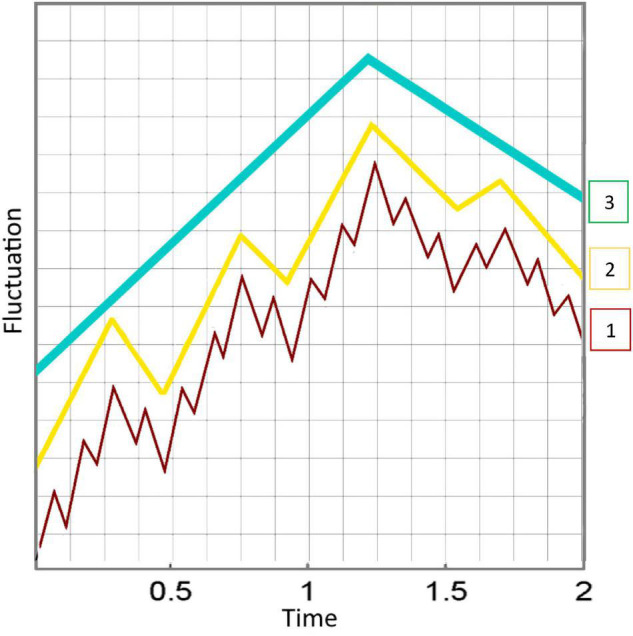
When coarse-grained representations consolidate, new levels of organization emerge. This results in a nested hierarchy within the conscious system, each level deriving from the ones underneath. The more regularities are identified in the environment, the higher the conscious process (3 > 2 > 1). The diagram shows three different levels, from fast variables with high fluctuations to emerging levels that are increasingly slower and generalized. The different wave patterns are superimposed, but here we have separated them for a more intuitive depiction of the emergent hierarchy. This may be misleading, as this increasing regularity is continuous, or graded, and not comprising of clear-cut levels.

Through the continuity of perception, an interaction history with the environment accumulates. As a result, the coarse-grained representations consolidate, constraining individual behavior and providing foundations of new levels of organization (see Figure 2). This results in a hierarchy within the conscious system, each level emerging from the ones underneath (Bayne et al., 2016). The more regularities are identified in the environment, the higher the conscious process. At higher levels of organization, more information is encoded about the environment in the system innately than through ongoing interaction with the environment, resulting in higher levels of consciousness. It is important to note that the different levels of consciousness in the hierarchy still answer to our definition of conscious experience: a subjective world of sensations, feelings, and thoughts that exhibit continuity over time, and can be turned outward at the world or inward at the self. In addition, we do not assert that these levels are clear-cut rungs on a ladder, but rather appear to smoothly transition from one to the next.

Such a hierarchical view implies that consciousness is graded in the sense that there are different degrees of consciousness (Overgaard and Overgaard, 2010; Bayne et al., 2016). It is not a matter of “all or none” – fully fledged consciousness or no consciousness at all; some systems or processes generate more consciousness than others. The emergence at any level of organization entails that consciousness can therefore be nested (Kirchhoff et al., 2018; Winters, 2020). From this perspective, we should not privilege any single level or object and define consciousness based on features of that object.

## Explaining the Aspects of Consciousness

Based on the simple principle of temporal integrity, the continuity of perception alone can lead to the emergence of many central phenomena associated with consciousness, without the need for any additional assumptions. This demonstrates the parsimony and explanatory power of our approach when considering phenomenological aspects. Indeed, any approach or model of consciousness should attempt to integrate mechanism and phenomenology (Hohwy and Seth, 2020). To begin with, the subjective sense of self, or “oneness,” is expressed not just in space but also in time: why do we attribute to our current self events and experiences from the past? Why do we feel that the person who went to sleep in the morning is the same person waking up? This persistent private point of view is referred to as subjectivity (Salti et al., 2019). It is the product of both temporal and spatial coherence. The temporal integrity of a conscious system traces a direct line between the system’s past states and the system’s current states. The integrated information, creating a unique and irreducible point of view, is dependent on these temporal dynamics. In this manner, our association with past selves is not an illusion, but the very foundation of conscious experience – propagating information across time. This is aligned with the observation that consciousness is continuous rather than discrete.

Meanwhile, the function and content of this information relates to one’s generative model of the environment. The individual does not just encode a model of the world, it *is* a model of the world (Ramstead et al., 2018). Our ongoing interaction with the environment, which is highly dependent on movement and the retainment of a single (and through movement, shifting) point of view, maps both our surroundings and their counterpart – ourselves. The regularities that we identify in this interaction tell us what and how we physically are. Properties of the self which are consistently reaffirmed are precisely those maintaining temporal integrity. This informational differentiation from the environment is what provides us with a clear sense of body boundaries.

This sense of oneness leads to another aspect of consciousness – self-consciousness, or the quality of being aware of ourselves, our existence, and our mental lives. It places the self as both the observer and the observed, such that one becomes the object of one’s own thoughts. Current models make a qualitative distinction between the mental processes that observe or access our thoughts and perceptions, and the thoughts and perceptions being observed or accessed (Block, 2001; Rosenthal, 2004). Our approach dispenses with the clear-cut separation. Instead, it perceives self-consciousness as the result of the hierarchical structure of conscious systems. As coarse-grained representations consolidate, new levels of organization emerge, reflecting the nested property of consciousness. The highest level of organization, which we associate with the conscious self, possesses maximal causal power. It identifies (and thus emerges from) patterns or regularities identified in lower levels of organization, much like lower levels of consciousness emerge from the recognition of slow variables in the environment. In this manner the mind can predict the behavior of lower levels and influence it. This implies that higher order states are rich conscious experiences that model the self along with the environment.

Finally, the awareness of the self as an entity differentiated from the environment and interacting with it leads to another aspect of human consciousness – volition. Human beings recognize their own volition, meaning we recognize an action as either what we intended to do or not (Haggard, 2019). It is the sense of having a will and a choice in how we behave. Reflecting the graded characteristic of consciousness, the difference between reflexes and deliberate decision-making is quantitative. Internally generated action does not mean that the action occurs out of nothing; volitional actions integrate many factors, such as multimodal sensory input, context, memory, and goals, so that it becomes hard to identify a single specific external trigger (Schüür and Haggard, 2011). Volitional behavior and experience occur in cognitive processes and stages that are “distanced” from primary sensory areas or the limbic system. For example, the prefrontal cortex integrates vast information, operates on slow-variables detached from the direct environment, and is endogenously driven (Fuster, 2001, 2004). A sense of freedom thus arises because actions are not governed by the immediacy of sensory input or motor output, but because of the range and complexity of the information that is integrated to cause the action.

## Additional Implications

Our information-theoretic view of consciousness has implications for the domain of consciousness and cognition. It allows us to reframe existing views on the nature of consciousness in a more parsimonious manner. First, cognitive processes considered unconscious are better viewed as dynamics with low causal power and temporal integrity. Unconscious mechanisms such as attentional mechanisms and primary sensory areas do not maintain information over time and are highly dependent on immediate input from their environment. The higher up the processing hierarchy we go, the slower the variables identified and the more resistant the representation produced [Kiebel et al., 2008; see Ballard (2015), Solms (2019)]: more microstates are consistent with the same macrostate. The conscious system becomes increasingly regular and indifferent to fluctuations, or noise, contributing to a higher degree of consciousness. Instead of postulating two opposed entities – conscious and unconscious processes – we are left with varying degrees of a single entity, namely consciousness.

By that token, our approach suggests simpler explanations for phenomena tackled by dominant theories of consciousness. To demonstrate, both the Global Neuronal Workspace Theory (GNWT) (Mashour et al., 2020) and Higher Order Theory (HOT) predict that consciousness is determined by prefrontal and parietal activity (Lau and Rosenthal, 2011). This requires the postulation of a multiplicity of dichotomic entities that are fundamentally distinct, such as conscious and unconscious perception, phenomenal and access consciousness, or first-order and higher-order representations, as well as specialized mechanisms for consciousness.

In contrast, our perspective suggests that processes preceding the activation of the prefronto-parietal network can still produce lower levels of consciousness, characterized by lower temporal integrity. The prefronto-parietal network simply drives an exponential increase in temporal integrity and causal power. It is activated by and its contents emerge from regularities identified in the information processed by lower levels in the hierarchy. Higher-order representations, or processes engaging with the global workspace, are accordingly endogenously determined, such that they are shaped by previous internal states rather than primary sensory and external input. From a phenomenological point of view, previous thoughts and sensations are the driving force behind new ones. These states are characterized by sustained thought, meta-cognition, introspection, and reportability. In this manner, the higher order mechanisms emerge naturally from primary principles and should not be separately assumed.

Another important implication of our view of consciousness relates to embodied cognition, the idea that the mind is not shaped just by the brain but by the entire body. The individuality of a biological system and its conscious experience are hard to decouple. It is no coincidence that we found the framework proposed by Krakauer et al. (2020) for the biological investigation of individuality to be highly applicable to consciousness. As Friston (2017) says, “I am, therefore I think.” As autonomy and differentiation from the environment emerge, so does consciousness. Given that consciousness is nested, it can be traced down to the level of the organism as a whole, albeit to a lesser degree. The privileged role attributed to the brain as “the seat of consciousness” is therefore simply due to the brain being responsible for the highest levels of temporal integrity (Ballard et al., 2013).

## Future Work and Conclusion

This article has defined and explained consciousness through the lens of information theory, as the propagation of information across time. Subjective experience is therefore an inevitable attribute of a system maintaining and in fact embodying across time a model of the environment by identifying regularities. We have demonstrated that such an account can help bridge mechanism and phenomenology, providing clues for varying aspects of consciousness, and ultimately offering a solution to the mind-body problem. In fact, one consequence is the blurred distinction between mind and body.

Future work should attempt to implement this approach in current theories of consciousness or even formalize it into a proper theory which would produce its own predictions on a high resolution in domains such as neuroscience, cognition, and psychology. The promise of parsimony would depend on the ability of this approach to account for concepts such as memory, attention, and executive functions, which often require the postulation of separate mechanisms whose relation to consciousness is unclear. On a larger scale, future discussion should revolve around how, by linking mechanism and phenomenology, this approach can inform neuropsychological phenomena, or even contribute to the formulation of parsimonious theories unifying biology and psychology, such as fostered by the concept of embodied cognition.

From this perspective, new approaches to neurocognitive research should take into account the temporal dimension, rather than considering the contents of consciousness as individuated, static states (Revach and Salti, 2021). In particular, the NCC program in the future should focus on the spatiotemporal dynamics of conscious perception (He, 2018). The suggestion that slow patterns of cortical potentials could be associated with consciousness (He and Raichle, 2009) resonates with and could be used to test the idea supported in this paper, that high levels of consciousness reflect the identification of slow, coarse-grained variables. King and Dehaene (2014) develop a method to generalize neural patterns across time that can be used to assess the evolving or changing complexity and stability of activity. Characterizing consciousness and its correlates could by such means take into account the information generated and its causal power over future states, linking these properties to phenomenology and performance [for example, see (Gu et al., 2003)]. The same methods can be used to study similarities and differences between human consciousness and that of other mammals, without having to rely on report and human neural structures.

These tools and other empirical methods will prove important in designing and testing applications of the framework we propose and some of the implications that go against dominant conceptions of consciousness. A few examples are the unconscious-conscious continuum as a function of temporal integrity, conscious experience as a process rather than a state, and the nested and hierarchical nature of consciousness arising from the encoding of increasingly slower variables.

The use of computer simulations can assist in understanding and affirming our predictions concerning the emergence of subjective properties (Koch and Tononi, 2011). By starting from fundamental principles of temporal integrity of information, artificial neural networks coupled with sensory apparatuses might progressively display behavior associated with consciousness, such as body boundaries and a sense of self or self-conceptualization.

## Data Availability Statement

The original contributions presented in the study are included in the article/supplementary material, further inquiries can be directed to the corresponding author.

## Author Contributions

DR wrote the manuscript under the supervision of MS. Both authors contributed to the article and approved the submitted version.

## Conflict of Interest

The authors declare that the research was conducted in the absence of any commercial or financial relationships that could be construed as a potential conflict of interest.

## Publisher’s Note

All claims expressed in this article are solely those of the authors and do not necessarily represent those of their affiliated organizations, or those of the publisher, the editors and the reviewers. Any product that may be evaluated in this article, or claim that may be made by its manufacturer, is not guaranteed or endorsed by the publisher.
